# Long-Term Exposure to Ceftriaxone Sodium Induces Alteration of Gut Microbiota Accompanied by Abnormal Behaviors in Mice

**DOI:** 10.3389/fcimb.2020.00258

**Published:** 2020-06-24

**Authors:** Zhongyi Zhao, Baoning Wang, Liyuan Mu, Hongren Wang, Jingjing Luo, Yuan Yang, Hui Yang, Mingyuan Li, Linlin Zhou, Chuanmin Tao

**Affiliations:** ^1^Department of Laboratory Medicine, West China Hospital, Sichuan University, Chengdu, China; ^2^Department of Microbiology, West China School of Basic Medical Sciences & Forensic Medicine, Sichuan University, Chengdu, China; ^3^Department of Laboratory Medicine, West China Second University Hospital, Sichuan University, Chengdu, China; ^4^State Key Laboratory of Oral Diseases, Sichuan University, Chengdu, China

**Keywords:** gut microbiota, emotional behaviors, ceftriaxone sodium, anxiety, depression, aggressive behavior

## Abstract

**Background:** Growing evidence points out that a disturbance of gut microbiota may also disturb the gut–brain communication. However, it is not clear to what extent the alteration of microbiota composition can modulate brain function, affecting host behaviors. Here, we investigated the effects of gut microbiota depletion on emotional behaviors.

**Methods:** Mice in the experimental group were orally administered ceftriaxone sodium solution (250 mg/ml, 0.2 ml/d) for 11 weeks. The open-field test and tail-suspension test were employed for the neurobehavioral assessment of the mice. Fecal samples were collected for 16s rDNA sequencing. The serum levels of cytokines and corticosterone were quantified using enzyme-linked immunosorbent assays. The immunohistochemistry method was used for the detection of brain-derived neurotrophic factor (BDNF) and c-Fos protein.

**Results:** The gut microbiota for antibiotic-treated mice showed lower richness and diversity compared with normal controls. This effect was accompanied by increased anxiety-like, depression-like, and aggressive behaviors. We found these changes to be possibly associated with a dysregulation of the immune system, abnormal activity of the hypothalamic-pituitary-adrenal axis, and an alteration of neurochemistry.

**Conclusions:** The findings demonstrate the indispensable role of microbiota in the gut–brain communication and suggest that the absence of conventional gut microbiota could affect the nervous system, influencing brain function.

## Introduction

Gut microbiota, known as a reservoir of bacteria, not only plays an essential role in host digestion and energy metabolism but shapes host immunity (Aleshukina, 2012; Antonopoulos and Chang, 2016; Thursby and Juge, 2017). Recently, evidence of its influence extends well-beyond the gut, many studies have begun to report that the gut microbiota may be associated with the development and progression of diseases affecting multiple organ systems such as liver, lung, and brain (Felix et al., 2018; Lee and Jayaraman, 2019; Yuan et al., 2019). Researchers believe that there is a potential connection between the gut and the central nervous system (CNS). Additional studies have defined this connection as a bi-directional communication covering multiple connections, such as immune response, the vagus nerve, and humoral components (Mayer et al., 2015). Recent evidence has unlocked a novel pivotal member, gut microbiota, which plays an important role in this communication. As a result, this concept, now known as the microbiota-gut-brain (MGB) axis, has been prompted and subsequently implicated in multiple disorders, such as digestive, neurological, and psychiatric diseases (Scriven et al., 2018; Iannone et al., 2019).

Antibiotics are one of the most commonly prescribed drugs worldwide. There has been an increasing concern that variations in the microbiota induced by antibiotics may have detrimental consequences for health (Kim et al., 2017). A growing body of evidence confirms the role of specific microbial compositions in the modulation of brain functions as well as host behaviors. To be specific, a complete absence of gut microbiota resulted in alteration of blood–brain barrier (BBB) permeability and brain neurochemistry with decreased social behaviors in mice (Braniste et al., 2014).

While the whole brain is vulnerable to external stimuli, two regions that influence stress responsivity and behavior have been considered as the most likely targets for gut microbiota (Luczynski et al., 2016). The first region is the amygdala, which seems to be involved in many forms of negative emotionality, including anxiety (Davis et al., 1994; Janak and Tye, 2015). After receiving input from disgust stimuli, the amygdala projects to the regions or sub-regions regulating anxious and defensive behaviors (Kovács, 2008). Usually, the activation of the amygdala is measured by c-Fos expression (Kovács, 2008). The second region, the hippocampus, is well-known as for emotion regulation. In this study, germ-free (GF) mice exhibited more anxiety-like behaviors, which were accompanied by higher brain-derived neurotrophic factor (BDNF) levels in the dentate region of the hippocampus (Sudo et al., 2004; Neufeld et al., 2011). Here, we explored the contribution of gut microbiota to the CNS via depleting bacteria with ceftriaxone sodium, a broad spectrum antibiotic.

## Materials and Methods

### Study Design

Male BALB/c mice (6–8 weeks; Institute of Laboratory Animals of Sichuan Academy of Medical Sciences, Sichuan, China) were maintained (ten mice per cage) under a specific-pathogen-free (SPF) condition at 22–26°C, 40–60% humidity, and 12-h light-dark cycle. The mice were given 1 week to acclimate. All mice were fed with adequate food and clean water. At the end of adaptive phase, all mice (initial weight 23.55 ± 1.49 g) were randomly divided into two groups (*n* = 20 for each group) and given either sterile saline solution (the control group was defined as the CT group, 0.2 ml/d) or ceftriaxone sodium solution (Qilu Pharmaceutical, Shandong, China) (the antibiotic group was defined as the AB group, 250 mg/ml, 0.2 ml/d) intragastrically once a day for 11 consecutive weeks (details about drug dosages is included in Supplementary Materials). Mice were housed by group (10 mice per cage) from the first day of gavage to avoid interference between different groups. A battery of behavioral tests was administrated weekly, with 1 h of rest between each test.

**Figure 1:**
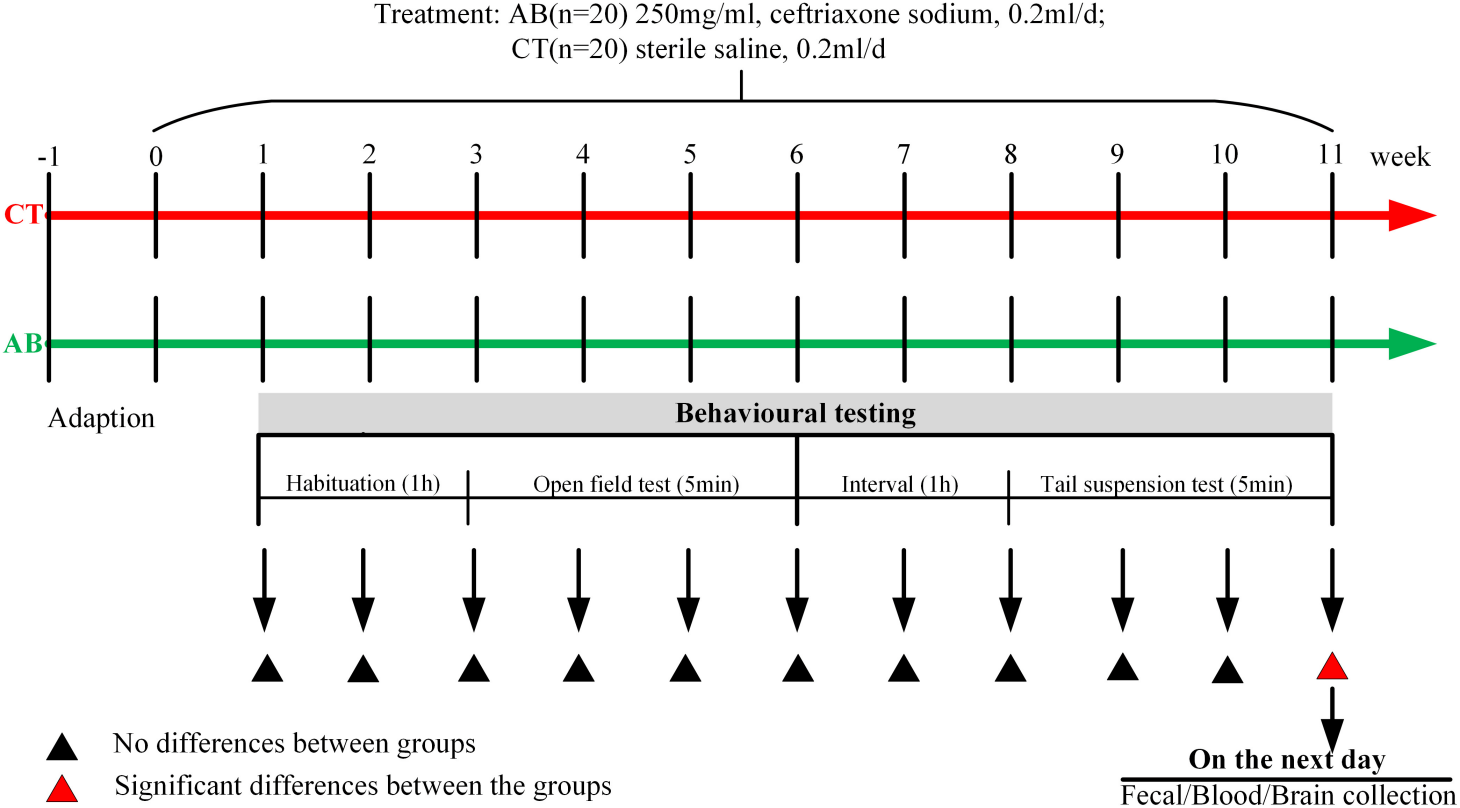
Study design. After seven days of adaptation, the antibiotic (AB) group was given ceftriaxone and the control (CT) group was given saline by gavage once a day. The open-field test (OFT) and tail-suspension test (TST) were given to mice once a week. After 11 weeks of oral gavage, significant behavioral differences were noted between the groups. On the second day after the last behavioral experiment was performed, mice were administered the final gavage exposure, and 1 h later, fresh blood and stool was sampled.

Eleven weeks after ceftriaxone treatment, the mice in the AB group exhibited a remarkable difference in behavioral parameters. On the second day after the last behavioral experiment was performed, the mice were administered the final gavage exposure, and 1 h later, fresh blood and stool was sampled. The mice were inspected daily for changes in appearance and body weight. All experiments followed the guidelines of the Chinese Council on Animal Care and were approved by the Animal Care Advisory Committee of Sichuan University, Sichuan, China. The experimental design was shown in Figure 1.

### Behavioral Tests

Two behavioral tests were carried out under following sequence (from 8 a.m. to 5 p.m.): open-field test (OFT) → tail-suspension test (TST). The OFT, which involves a low stress level, preceded the TST, which involves a high stress level (Di et al., 2017). Prior to each behavioral test, mice were habituated for at least 1 h to the testing room (Champagne-Jorgensen et al., 2020). The lighting condition was set at 15 lux for all behavioral tests (Dere et al., 2004).

#### The Open-Field Test

The equipment of the OFT was composed of a square arena 100 × 100 cm with 40 cm walls. The floor was subdivided into a center and periphery compartment with 25 squares. Mice were placed alternatively in the open field for at least 30 min and allowed to explore undisturbedly before the first test (Champagne-Jorgensen et al., 2020). In the formal test, mice were placed singly in the center of the open field and allowed to freely explore for 5 min. Relevant parameters (the total distance, the total time in the periphery, and center of the open field) were recorded by a video monitor. At the end of the test, mice were sent back to their home cages, and the test box was cleaned with 70% ethyl alcohol and air dried. The OFT has been proven to be efficient in detecting anxiety and selecting anxiolytic drugs (Kraeuter et al., 2019).

#### The Tail-Suspension Test

Mice were suspended in an upside-down position by the tail, so that they could not escape or touch nearby surfaces. The rationale for the test is that mice are under enormous stress, and, if they don't have the desire to live, they will develop a motionless posture quickly and maintain it for a longer period. The total duration of quiescence and activity during 5 min was scored, respectively (Młyniec and Nowak, 2012).

### Gut Microbiota Analysis

A TIANamp Bacteria DNA Kit (TIANGEN, China) was used to extract fecal DNA. Then, the extraction was eluted using elution buffer and stored at −80°C until PCR amplification detection by LC-Bio (Hangzhou, China). The V3-V4 region of the prokaryotic 16S rRNA gene was amplified with primers 338F (5′-ACTCCTACGGGAGGCAGCAG-3′) and 806R (5′-GGACTACHVGGGTWTCTAAT-3′) (Fadrosh et al., 2014). The detailed operation was performed as described previously (Li et al., 2019).

### Serum Cytokine Assay

Cytokines secretion is usually induced in an inflammation or infection. Except for their effects on immunity, cytokines can also affect brain function and modulate host behaviors (Köhler et al., 2018). Research has suggested that serum IL-6 and IL-10 levels are putative biomarkers for several mood disorders (Wiener et al., 2019). Here, fresh blood was collected in sterile tubes, coagulated at room temperature, and centrifuged at 1000 × g for 10 min after the last ceftriaxone sodium treatment. The serum was stored at −70°C for later analysis. IL-6 and IL-10 were quantified by enzyme-linked immunosorbent assay (ELISA) (Neobioscience, Shenzhen Xinbosheng Biotechnology Co., Ltd, China). The detection limit of the assay was about 1 pg ml^−1^. According to the manufacturer's protocol, the assay was performed in triplicate.

### Serum Corticosterone Assay

Corticosterone is the end product of the hypothalamus-pituitary-adrenal (HPA) axis in rodents. Rising corticosterone levels suggest increases in HPA axis activity (Hiroshi et al., 2006). Serum corticosterone was measured by ELISA (Cusabio,Wuhan Huamei Biotechnology Co., Ltd, China). The detection limit of the assay was about 1 ng ml^−1^. The assay was performed in triplicate according to the manufacturer's protocol.

### Immunohistochemistry

The immunohistochemistry was used to assess expression of brain-derived neurotropic factor (BDNF) in the hippocampus and c-Fos in the amygdala. The whole process consisted of brain collection, sectioning, and immunolabeling. The details referred to previous literatures (Gareau et al., 2011).

Each maker was quantified by staining intensity and extent. We scored the staining intensity as follows: negative, weak, moderate, and strong (on a scale of zero to four). The staining extent was divided into five grades according to the percentage of positive cells in the region: negative, 0–25, 26–50, 51–75, and 76–100% (on a scale of zero to four) (Liu et al., 2011).

Semi-quantification of BDNF was calculated by multiplying the intensity score and fraction score in the CA1, CA3, and DG (dentate gyrus) regions of the hippocampus (Olympus, Tokyo, Japan, BX53). Similarly, semi-quantification of c-Fos was performed by calculating the intensity score and fraction score in the CeC, CeL, and CeM regions of the amygdala. The immunohistochemical analysis was performed blind.

### Statistical Analysis

Data were expressed as the mean ± standard deviation or median (IQR) and analyzed by one-way ANOVA or Wilcoxon rank sum test in SPSS 22.0 software (SPSS Inc., Chicago, IL, USA). *P*-values less than 0.05 were considered statistically significant.

## Results

### The Effect of Ceftriaxone Treatment on Body Weight

**Figure 2:**
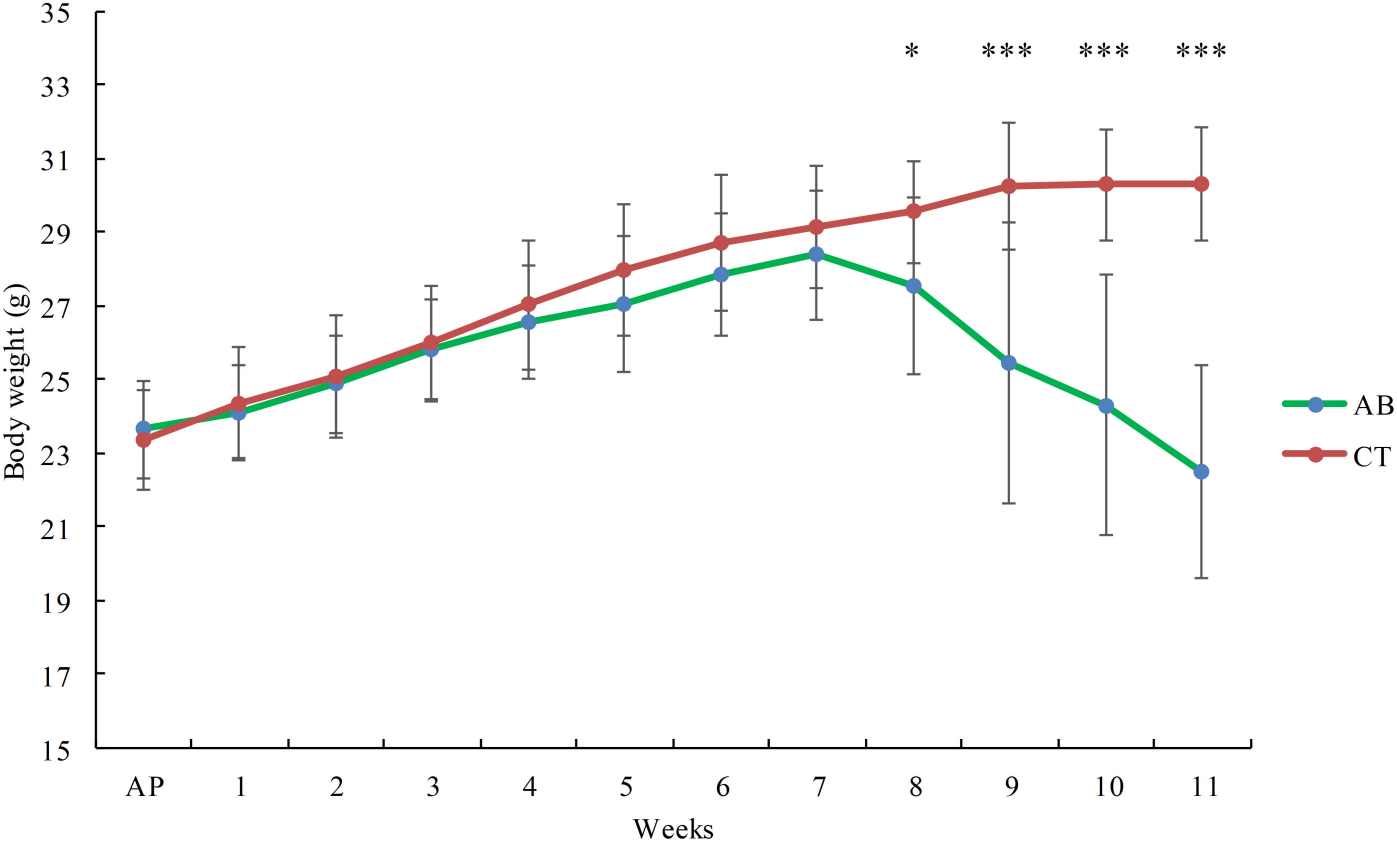
Effect of ceftriaxone on body weight. AB, ceftriaxone administration for 11 weeks (*n* = 14); CT, saline treatment for 11 weeks (*n* = 18). AP: adaptive phase. *P < 0.05, ***P < 0.001.

Mice in the AB group gained less weight than the CT group, with this difference increasing progressively over time. After gavage for 7 weeks, the weight of the AB group was significantly lower than that of the CT group (*p* < 0.05) (Figure 2).

### The Effect of Ceftriaxone Treatment on Mice Behaviors

#### Mice Treated With Ceftriaxone Sodium Exhibited Anxiety-Like Behaviors

**Figure 3:**
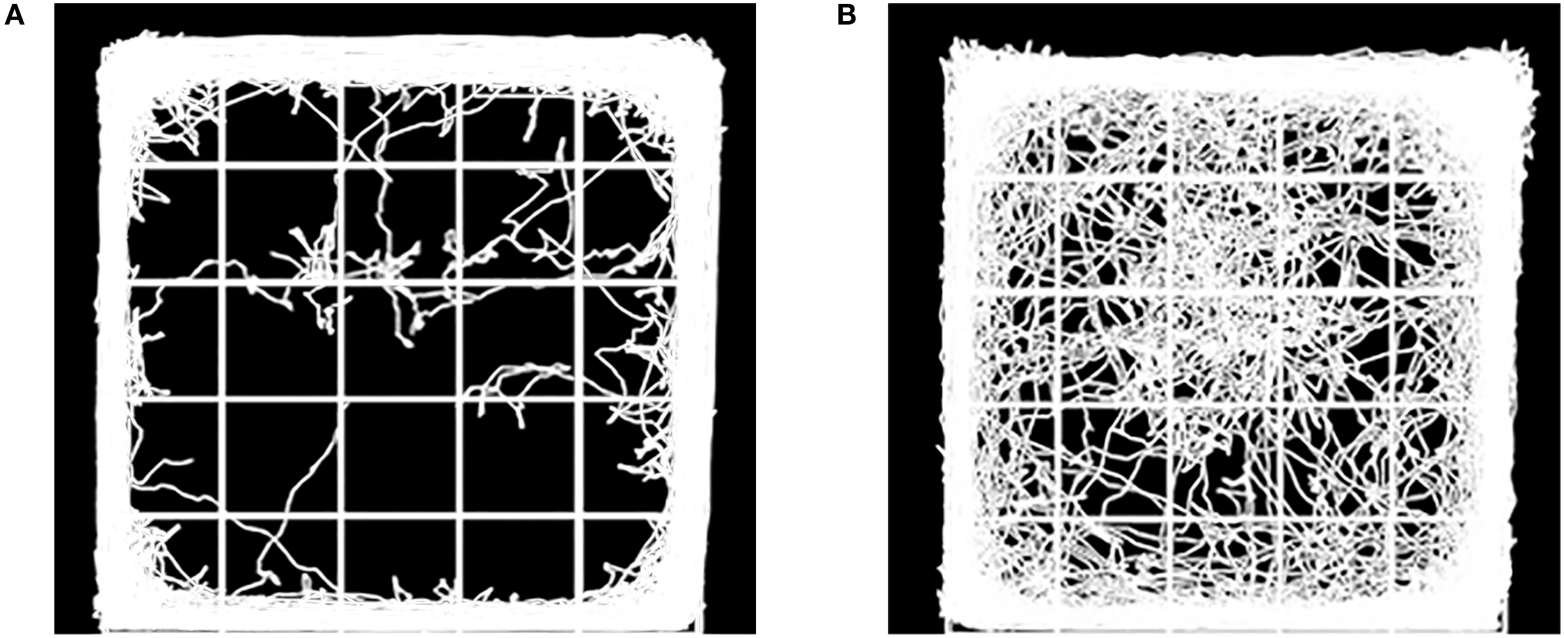
Diagram showing movement of **(A)** AB group mice and **(B)** CT group mice in the open field. AB, antibiotic group (*n* = 14); CT, control group (*n* = 18).

**Figure 4:**
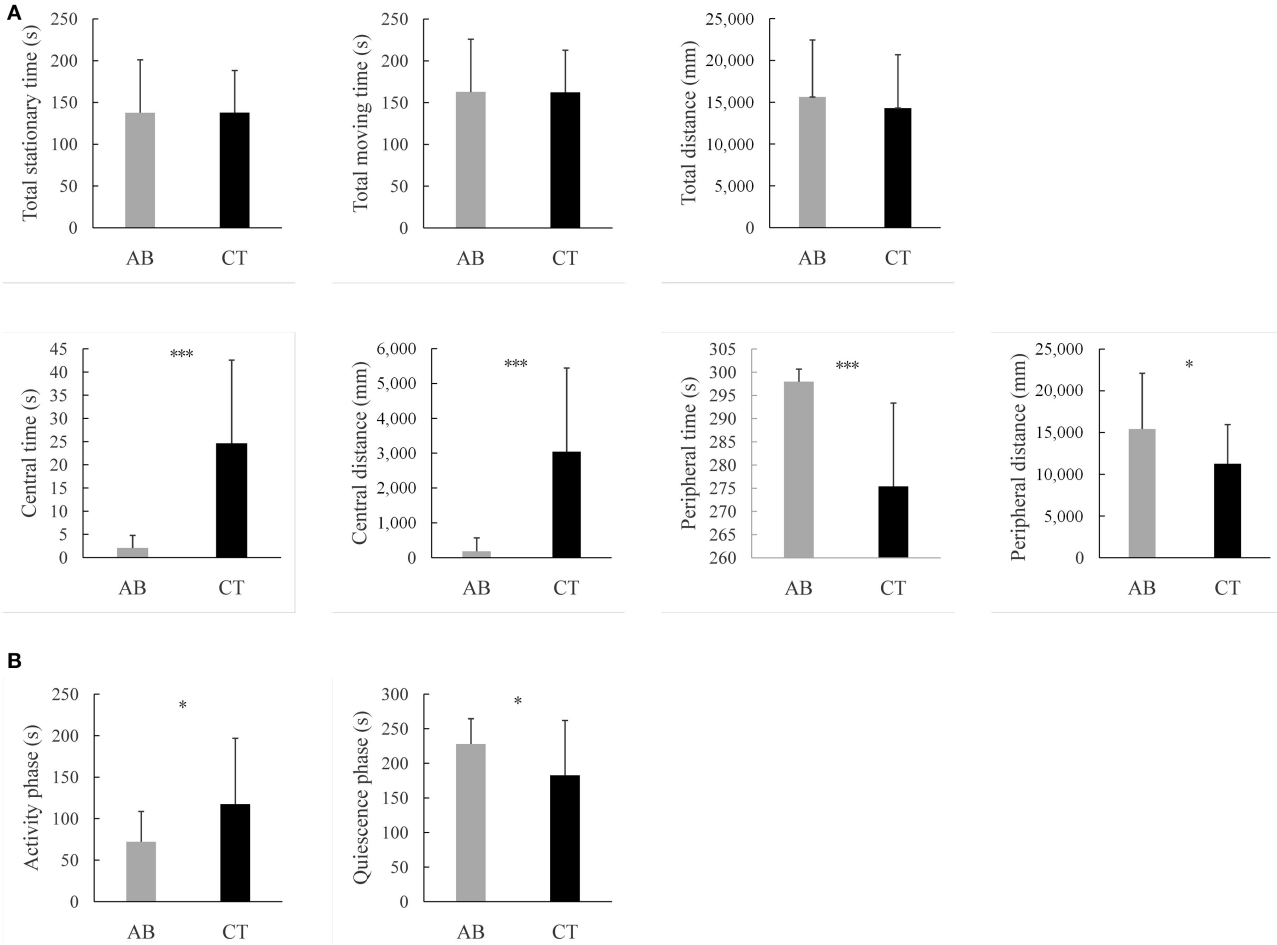
Results of behavioral tests **(A)** in the open field test, **(B)** in the suspension tail test. AB, antibiotic group (*n* = 14); CT, control group (*n* = 18). *P < 0.05, ***P < 0.001.

OFT is usually performed to assess locomotor activity and exploratory behavior (Kraeuter et al., 2019). The former was represented by the total distance traveled throughout the 5 min, and no differences were observed between the two groups. Previous studies suggest mice prefer staying close to the walls and travel more in the periphery field can be described as showing signs of anxiety (Crawley, 1985). Comparatively, mice with lower anxiety tend to spend more time in the central field. In this study, the AB group spent less time in the center as compared to the CT group (p < 0.001) after gavage for 11 weeks. Meanwhile, the AB group reduced movement in the center (p < 0.001) (Figures 3, 4A). For details of the 11-week behavioral data analysis, see Supplementary Figure 1.

#### Mice Treated With Ceftriaxone Sodium Exhibited Depression-Like Behaviors

TST was often used for evaluating the ability to cope with a stressful situation (Kraeuter et al., 2019). Decreased duration of activity is considered a sign of depressive behavior (Castagne et al., 2011). In the test, the AB group showed decreased activity during the 5 min and stopped escaping earlier than the CT group (*p* < 0.05) after gavage for 11 weeks (Figure 4B).

#### Mice Treated With Ceftriaxone Sodium Exhibited High Aggressive Behavior

Eight weeks after ceftriaxone administration, visible injuries were observed in the AB group, suggesting that aggressive behaviors had occurred. Four mice from the AB group were excluded from the experiment 10 weeks later due to serious injuries influencing mobility. In contrast, the CT group did not get injured throughout the experiment.

### The Effect of Ceftriaxone Treatment on Gut Microbiota Composition

**Figure 5:**
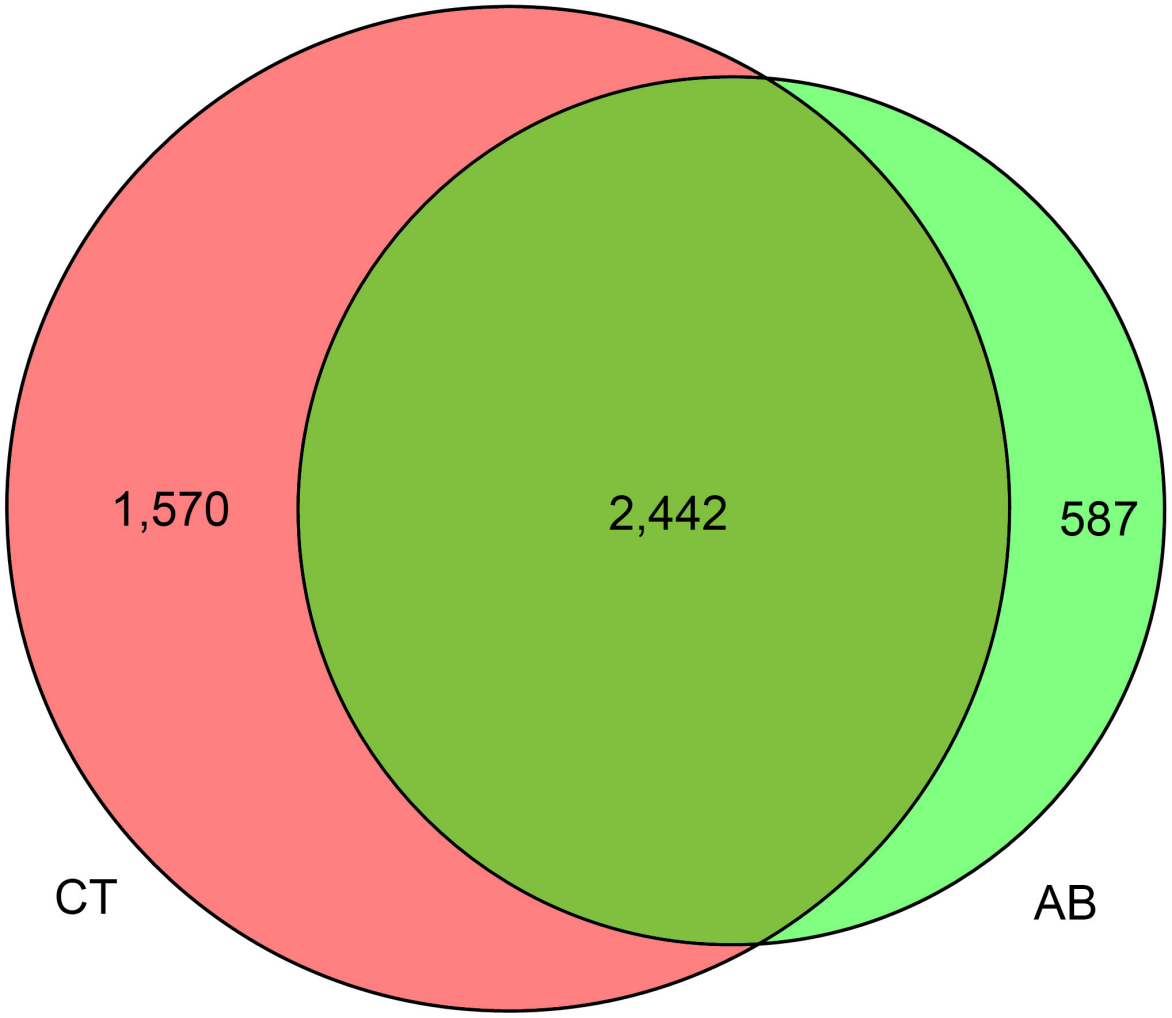
Venn diagram of fecal bacteria. AB, antibiotic group (*n* = 14); CT, control group (*n* = 18).

**Figure 6:**
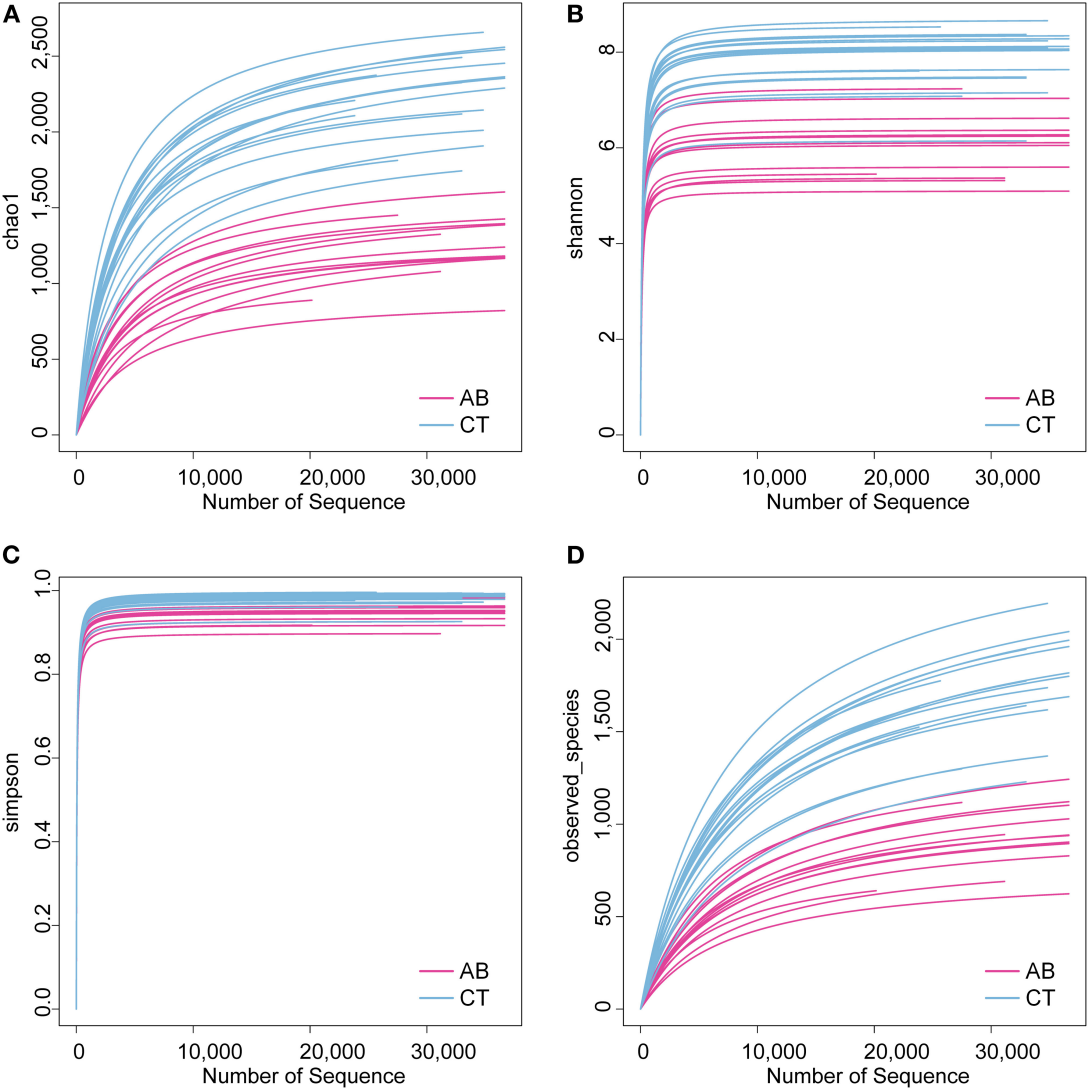
Alpha diversity analysis for gut microbiota. **(A)** Chao1 curves for each group. **(B)** Shannon-Wiener curves for each group. **(C)** Simpson curves for each group. **(D)** Observed_species curves for each group. AB, antibiotic group (*n* = 14); CT, control group (*n* = 18).

**Figure 7:**
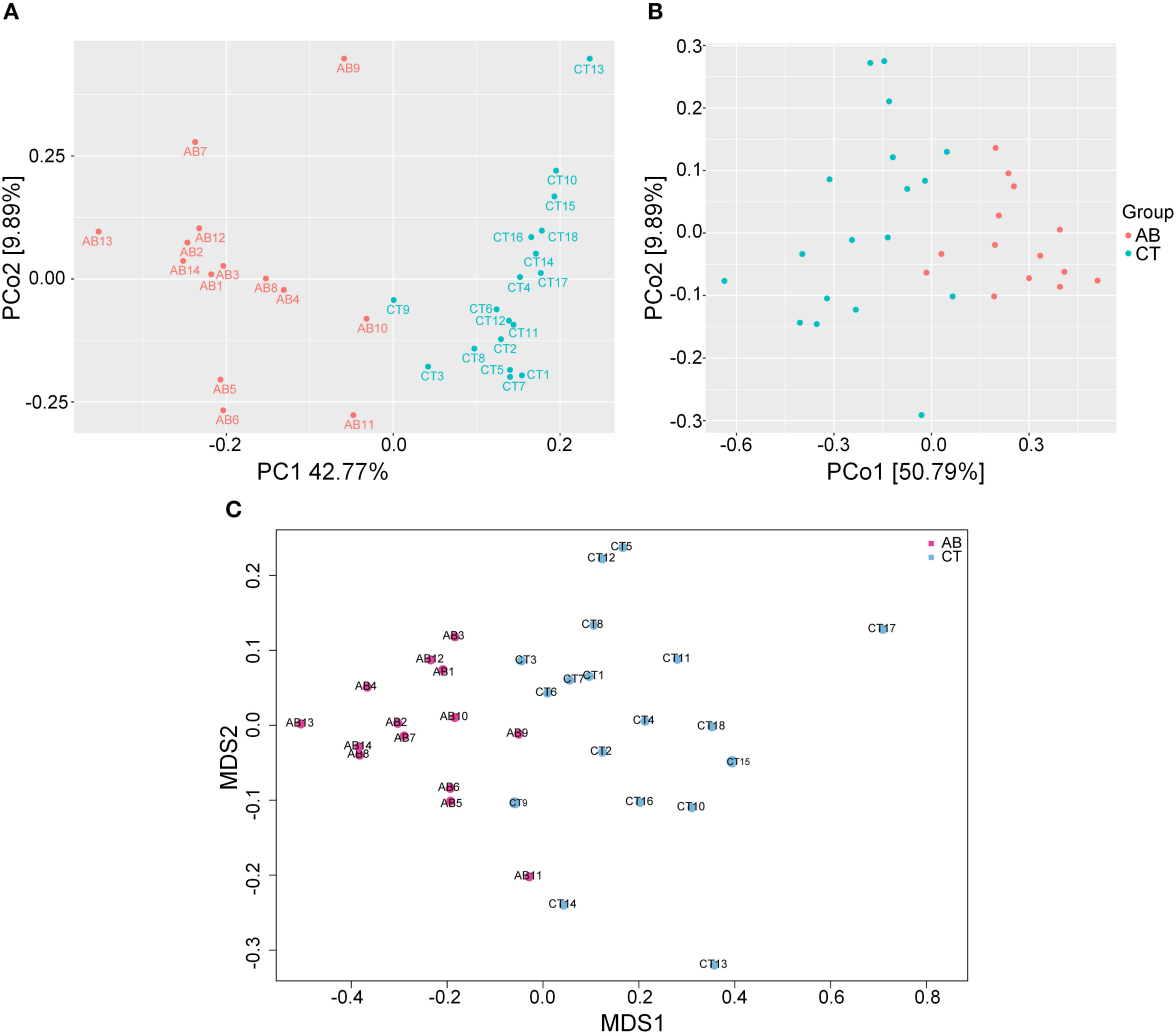
Beta diversity analysis for gut microbiota. **(A)** PCA plot of weighted UniFrac distances between samples. **(B)** PCoA plot of weighted UniFrac distances between samples. **(C)** MDS plot of weighted UniFrac distances between samples. AB, antibiotic group (*n* = 14); CT, control group (*n* = 18).

16S rDNA sequencing was used to identify alterations in gut microbiota after gavage for 11 weeks. Ceftriaxone administration induced a significant change in gut microbiota diversity. For the Venn diagram (Figure 5), the number of shared and unique OTUs indicate a similarity and difference of gut microbiota between groups, respectively (Ren et al., 2018). Based on this, there were 587 OTUs specific to the AB group and 1,570 specific to the CT group, accounting for 19.38 and 39.13% of the total OTU richness, respectively. All samples shared 2,442 OTUs at 97% similarity. The alpha diversity analysis revealed that the AB group had lower species diversity, richness, and evenness than that of the CT group by plotting Chao1, Shannon, Simpson, and Observed_species curves (Figure 6). A strong antibiotic effect was observed in beta diversity analysis. The PCA plot showed an appreciable separation between the two groups, indicating they had low similarity in gut microbiota composition. Likewise, the PCoA plot and MDS plot indicated the microbiota of AB group clustered separately from CT group (Figure 7).

**Figure 8:**
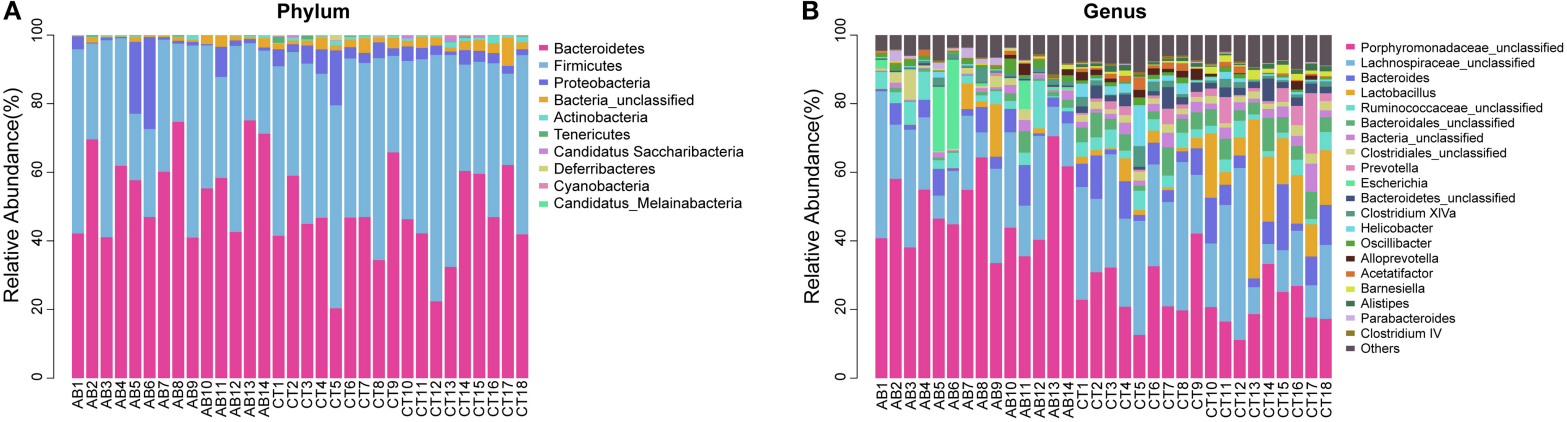
Composition abundance of gut microbiota **(A)** at the phylum level and **(B)** the genus level. AB, antibiotic group (*n* = 14); CT, control group (*n* = 18).

**Figure 9:**
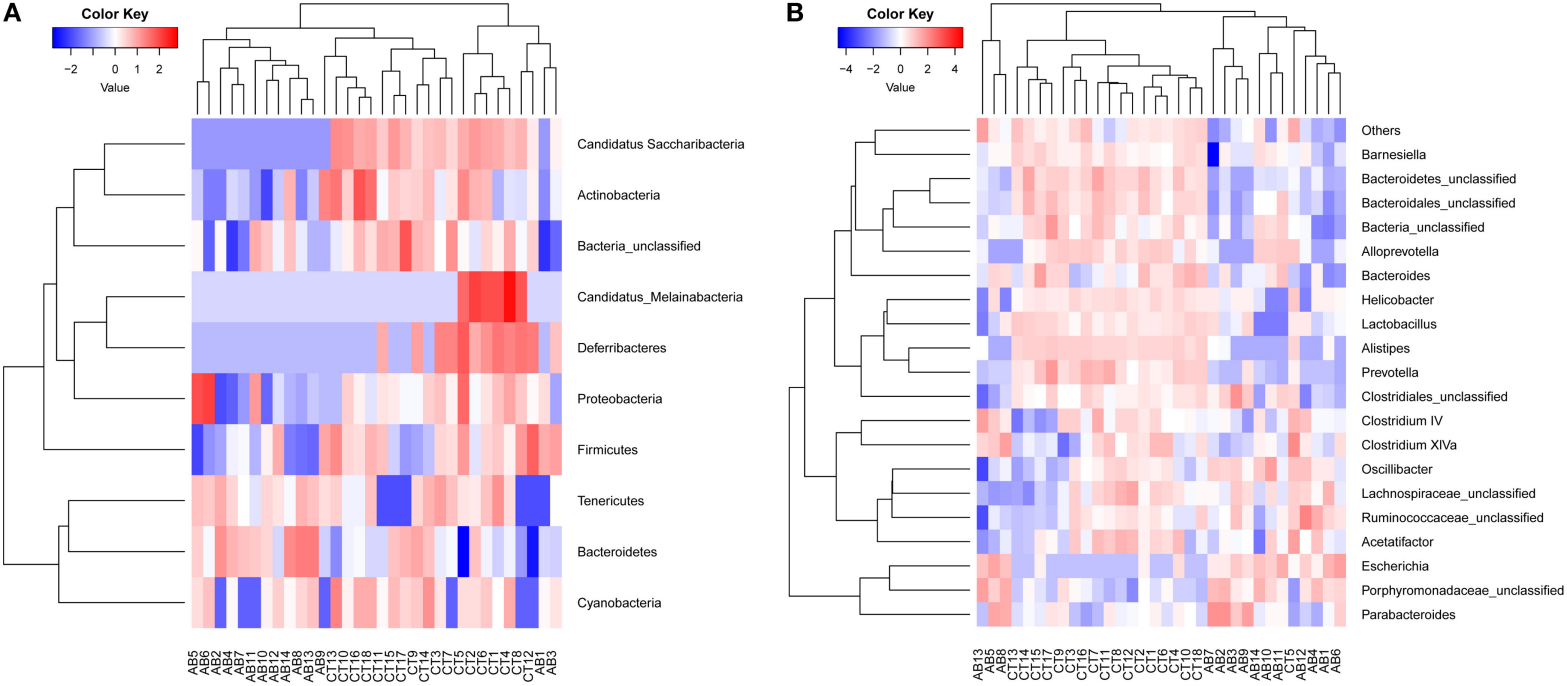
Heat maps of gut microbiota **(A)** at the phylum level and **(B)** the genus level. Red and blue colors indicate high and low values of the percent of reads classified at that rank. AB, antibiotic group (*n* = 14); CT, control group (*n* = 18).

According to abundance analysis, the dominant phyla in AB and CT groups were *Bacteroidetes* and *Firmicutes*, while the relative abundance of *Firmicutes* was lower in the AB group than that in CT group. Significant abundance differences were observed in the following phyla: *Proteobacteria* increased while five phyla decreased (*Firmicutes, Actinobacteria, Candidatus Saccharibacteria, Deferribacteres*, and *Candidatus_Melainabacteria*) in the AB group (Figure 8A, Table 1). At the genus level, ceftriaxone increased the proportion of *Proteobacteria, Porphyromonadaceae_unclassified, Escherichia*, and *Parabacteroides*, while *Lactobacillus, Acetatifactor, Bacteroidetes_unclassified, Barnesiella, Helicobacter, Prevotella, Alistipes*, and *Bacteroidales_unclassified* declined (Figure 8B, Table 2). Ten phyla and 21 genera were clustered by heatmaps, which demonstrated the relative abundance of species in different samples. In the CT group, the samples got closer to each other, indicating a higher similarity among them (Figure 9).

**Table 1:** Relative abundance of gut microbiota at the phylum level.

**Phylum**	**AB (%)**	**CT (%)**	***P*-value**
*Candidatus_Saccharibacteria*	0.00	0.27	0.006^**^
*Actinobacteria*	0.34	0.81	0.006^**^
*Deferribacteres*	0.00	0.15	0.008^**^
*Bacteria_unclassified*	1.17	2.62	0.005^**^
*Candidatus_Melainabacteria*	0.00	0.01	0.024^*^
*Proteobacteria*	4.89	4.13	0.034^*^
*Firmicutes*	36.51	46.12	0.047^*^
*Bacteroidetes*	56.97	45.58	0.064
*Cyanobacteria*	0.01	0.09	0.097
*Tenericutes*	0.11	0.23	0.379

*AB, antibiotic group (n = 14); CT, control group (n = 18)*.

* *P < 0.05*,

** *P < 0.01*.

**Table 2:** Relative abundance of gut microbiota at the genus level.

**Genus**	**AB (%)**	**CT (%)**	***P*-value**
*Porphyromonadaceae_unclassified*	49.13	23.43	0.000^***^
*Lachnospiraceae_unclassified*	20.49	24.57	0.335
*Bacteroides*	4.32	7.07	0.082
*Lactobacillus*	1.86	9.23	0.028^*^
*Ruminococcaceae_unclassified*	4.21	3.45	0.408
*Bacteroidales_unclassified*	1.31	4.89	0.000^***^
*Bacteria_unclassified*	1.17	2.62	0.007^**^
*Clostridiales_unclassified*	1.80	2.03	0.673
*Prevotella*	0.10	3.14	0.010^*^
*Escherichia*	4.16	0.00	0.038^*^
*Bacteroidetes_unclassified*	0.32	2.65	0.000^***^
*Clostridium XlVa*	1.21	1.47	0.571
*Helicobacter*	0.24	1.93	0.029^*^
*Oscillibacter*	1.31	0.84	0.125
*Alloprevotella*	0.43	1.10	0.074
*Acetatifactor*	0.44	1.07	0.033^*^
*Barnesiella*	0.30	1.11	0.002^**^
*Alistipes*	0.00	1.00	0.000^***^
*Parabacteroides*	0.95	0.15	0.004^**^
*Clostridium IV*	0.40	0.40	0.953

*AB, antibiotic group (n = 14); CT, control group (n = 18)*.

* *P < 0.05*,

** *P < 0.01*,

*** *P < 0.001*.

### The Effect of Ceftriaxone Treatment on Serum Cytokines and Corticosterone

**Figure 10:**
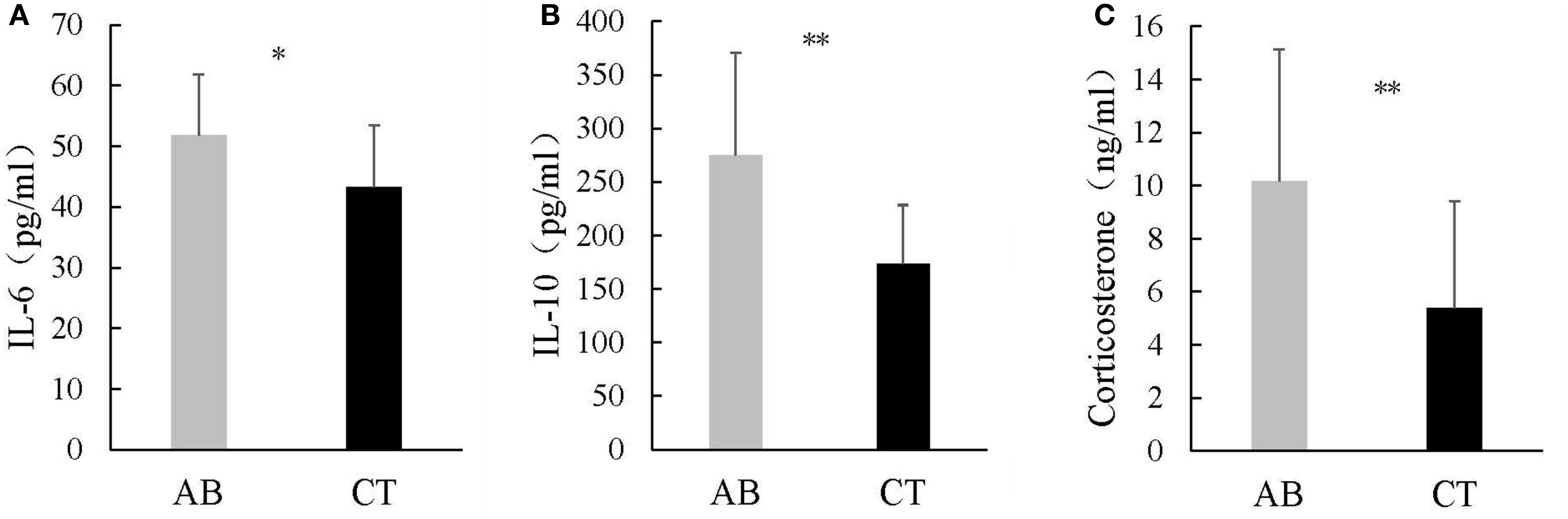
Concentrations of **(A)** IL-6, **(B)** IL-10, and **(C)** corticosterone in serum. AB, antibiotic group (*n* = 14); CT, control group (*n* = 18). *P < 0.05, **P < 0.01.

Ceftriaxone induced increased IL-6 and IL-10 in the AB group (IL-6: 51.82 ± 9.99 pg/ml and 43.21 ± 10.18 pg/ml for AB and CT, respectively) (IL-10: 274.81 ± 95.59 pg/ml and 173.12 ± 55.31 pg/ml for AB and CT, respectively) (Figures 10A,B). In addition, serum corticosterone was significantly higher in the AB group than in the CT group (10.16 ± 4.97 ng/ml and 5.39 ± 4.03 ng/ml for AB and CT, respectively) (Figure 10C).

### The Effect of Ceftriaxone Treatment on Hippocampal Cell Proliferation and Neural Activity

**Figure 11:**
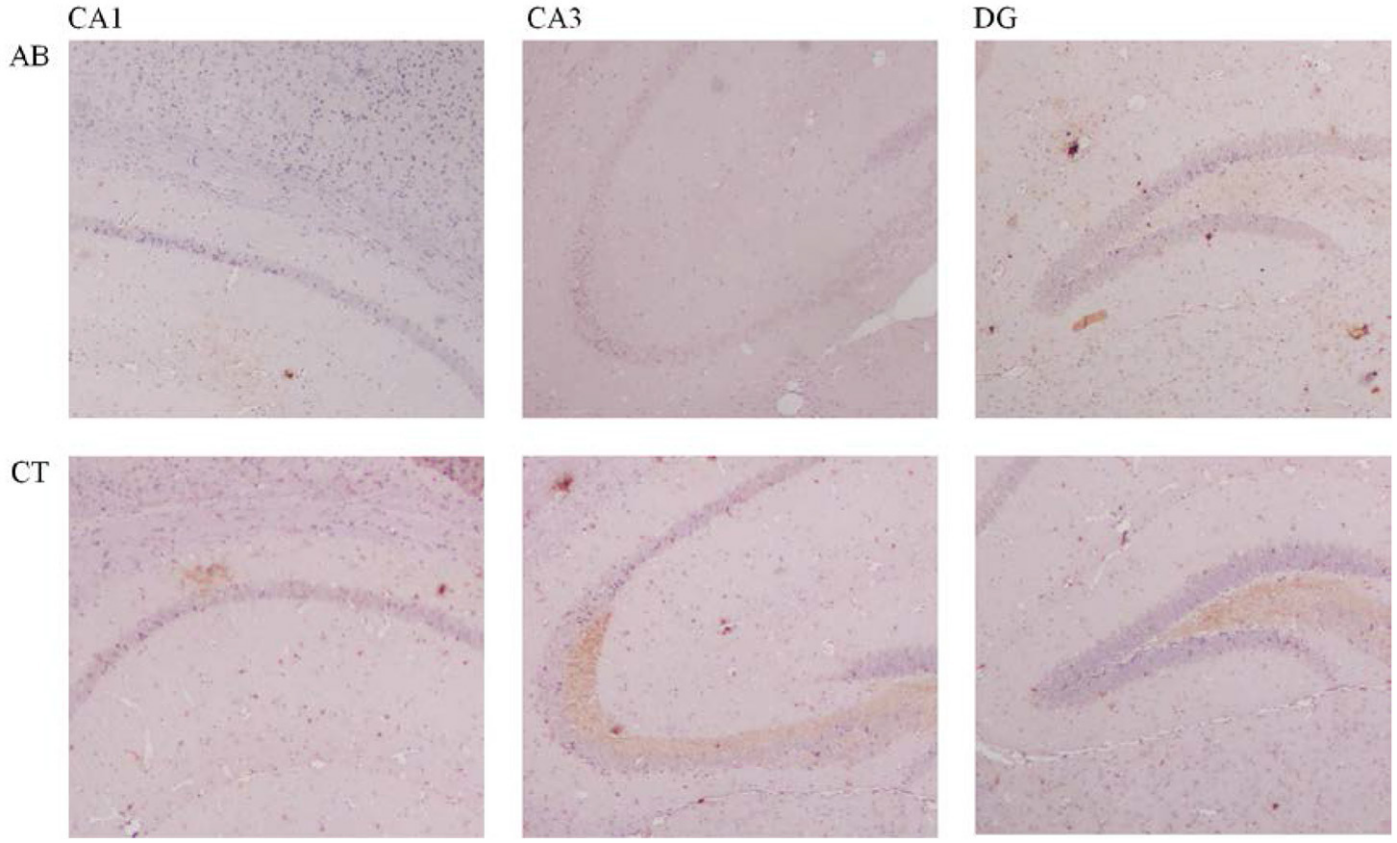
Immunohistochemical results of BDNF in the hippocampus of mice (×400). AB, antibiotic group (*n* = 14); CT, control group (*n* = 18). CA1, field CA1 of hippocampus; CA3, field CA3 of hippocampus; DG, dentate gyrus.

**Figure 12:**
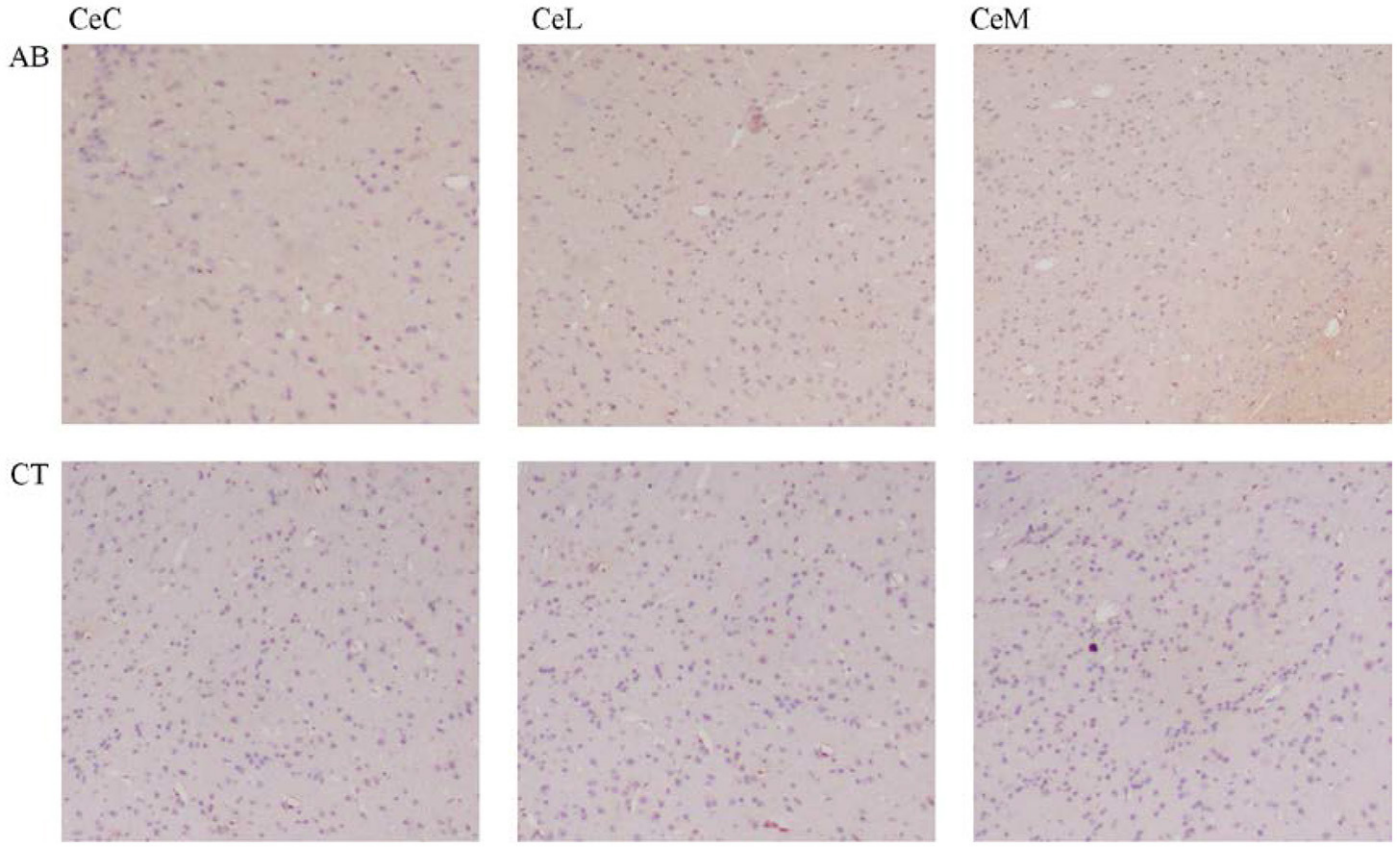
Immunohistochemical results of c-Fos in the amygdala of mice (×400). AB, antibiotic group (*n* = 14); CT, control group (*n* = 18). CeC, central amygdaloid nucleus, capsular part; CeL, central amygdaloid nucleus, lateral division; CeM, central amygdaloid nucleus, medial division.

A slight decrease of BDNF in the CA1, CA3, and DG regions of the hippocampus was observed in the AB group compared to the CT group (Figure 11, Table 3). Meanwhile, c-Fos expression increased in the amygdala of the AB group without a statistically significant difference (Figure 12, Table 3).

**Table 3:** Expression of BDNF in the hippocampus and expression of c-Fos in the amygdala.

		**BDNF**			**c-Fos**		
**Group**	***n***	**CA1**	**CA3**	**DG**	**CeM**	**CeL**	**CeC**
AB	14	1 (1.00–3.25)	3 (2.00–6.75)	3 (1.75–4.00)	6 (3.00–7.50)	6 (3.00–7.50)	2 (0.50–5.00)
CT	18	2 (1.00–4.00)	4 (2.25–8.25)	3 (2.00–4.00)	0 (0.00–4.50)	0 (0.00–4.50)	1 (0.00–9.00)
*P*-value		0.350	0.586	0.884	0.076	0.076	0.892

*AB, antibiotic group; CT, control group; CA1, field CA1 of hippocampus; CA3, field CA3 of hippocampus; DG, dentate gyrus; CeM, central amygdaloid nucleus, medial division; CeL, central amygdaloid nucleus, lateral division; CeC, central amygdaloid nucleus, capsular part*.

## Discussion

The gut, a vulnerable but vital organ, is affected by different factors easily. Antibiotics are one of the common causes leading to gut disturbance, especially given the broad spectrum of antibiotics. Consistently, little is known about adverse effects of these antibiotics on health except for drug resistance. But, recently, medications with antibiotic have been reported to enhance the risk of allergies, inflammatory bowel diseases, obesity, and even mental diseases (Harris and Baffy, 2017; Torres-Fuentes et al., 2017; Guo J. et al., 2019; Slykerman et al., 2019). Also, some investigators suggest that abnormal gut microbiota or some intestinal infections may be responsible for a series of metabolism or immunity-related diseases (Wang and Wang, 2016). The impact of intestinal dysbacteriosis induced by antibiotics on brain functions and behaviors piques interest and is yet to be elucidated. Therefore, this study was designed to determine whether long-term ceftriaxone exposure altered gut microbiota and thus affected host behaviors.

Ceftriaxone administration caused significant weight loss in the study. This is consistent with the previous finding that the weight gain of mice was delayed significantly following the ceftriaxone treatment (Miao et al., 2020). However, it was contradictory with previous findings that antibiotics result in weight gain in the animal production system (Angelakis, 2017). The disparate findings imply that the growth of animals may be impacted by the dosage, intervention time and, above all, types and properties of antibiotics.

Ceftriaxone could result in a significant gut microbiota dysbiosis by killing most of the normal flora and providing the living space for other potential pathogens (Cheng et al., 2019). The gut microbiota of mice was altered greatly in quantity and quality by the oral administration of ceftriaxone in this study. Similar to the result, other studies confirmed that oral ceftriaxone significantly decreased the quantity of fecal microbiota (Cheng et al., 2017, 2019; Guo et al., 2017; Miao et al., 2020). At the phylum level, the microbiota diversity of the AB group decreased, *Proteobacteria* became a dominant phylum, and the abundance of *Bacteroidetes, Firmicutes, Actinobacteria*, and *Deferribacteres* decreased. This result is supported by studies that ceftriaxone could characteristically decrease the alpha-diversity of the fecal microbiota accompanied with more *Proteobacteria* and less *Bacteroidetes* (Cheng et al., 2017, 2019; Miao et al., 2020). In some dysbiosis and related diseases, an increased *Proteobacteria* is perceived as a diagnostic characteristic since it is closely related to colon epithelial oxygenation as well as the disruption of the gut anaerobic environment (Zhu et al., 2013; Shin et al., 2015; Miao et al., 2020). *Firmicutes* has become a controversial strain as some studies identified an increase in *Firmicutes* after ceftriaxone treatment, but others have demonstrated a declined *Firmicutes* in the ceftriaxone group (Cheng et al., 2017, 2019; Miao et al., 2020). Evidence from clinics has suggested that patients with depression often have decreased *Firmicutes* (Huang et al., 2018). Experimental findings further revealed that decreased *Firmicutes* led to a reduction in short-chain fatty acids, which are an important physiological basis for low-level inflammation during depression (Huang et al., 2018). *Bacteroidetes*, as an important microbe for short-chain fatty acids, almost disappeared from the feces of the mice during exposure to ceftriaxone (Miao et al., 2020). Significant alteration of fecal microbiota was also observed at the genus level: *Porphyromonadaceae, Escherichia*, and *Parabacteroides* dominated the gut microbiota of the AB group mice, while *Lactobacillus, Clostridiales, Acetatifactor, Bacteroidetes, Barnesiella, Helicobacter, Prevotella, Bacteroidales*, and *Alistipes* were lowered. In line with this, some researchers have proposed that decreased *Barnesiella* after ceftriaxone gavage is a common and sensitive gut microbiota of the BALB/c mice and can be used as an indicator for assessing the balance of the gut microbiota (Zhao et al., 2013). *Bacteroidetes* is closely associated with digestion and interacts with the host's immune system, affecting the growth of other bacteria (Karlsson et al., 2011). In addition, an increase in *Escherichia* prevalence after oral antibiotic treatment has been reported for vancomycin and imipenem (Stokes, 1949), amoxicillin, bismuth (Dawes and Foster, 1956), and metronidazole (Paege and Gibbs, 1961). It is difficult to discern whether an increase in *Escherichia* could be beneficial or harmful as *Escherichia* is both a commensal and pathogenic inhabitant of a host's gastrointestinal tract. But most of the time, *Escherichia* is considered a potential pro-inflammatory bacteria (Liu et al., 2019). Of particularly note, increased *Porphyromonadaceae* associates with mental deficits and cognitive disorders as well as anxiety-like behaviors in mice (Scott et al., 2017). *Lactobacillus* is known as a protective species against long-lasting metabolic disturbances and prevents gut dysbiosis, but was suppressed by ceftriaxone (Robles-Vera et al., 2018). Researchers also discovered that elevated *Parabacteroide* relates to the etiology of depression (Cheung et al., 2019). These results indicate, once again, that different bacteria may be involved in different functions or biological pathways.

Alterations in gut microbiota were accompanied by behavioral changes in the mice, including anxiety-like, depression-like, and aggressive behaviors. These behavioral changes cannot necessarily be a result of the direct toxic effect of ceftriaxone on the brain, since ceftriaxone is a non-absorbable antibiotic and usually given by injection. Previous studies have demonstrated the complex interaction between gut microbiota and the CNS; this is what is known as the MGB axis (Wang and Wang, 2016). Animal experiments support that absence or change in gut microbiota affects the HPA axis answering to stress, anxiety, and relevant behavior (Koopman and El Aidy, 2017; Lach et al., 2018; Chen et al., 2019). In addition, the rodents infected with intestinal pathogens showed anxiety-like behaviors, which can be partly explained by the activation of vagal afferents (Klarer et al., 2014). In one study of GF BALB/c with a high-anxiety level and NIH Swiss mice with a high exploratory ability, when the two groups exchanged each other's microbiota, the donor behavioral characteristics could be reproduced in recipients (Crumeyrolle-Arias et al., 2014). On the other hand, clinical trials suggest treatment with probiotics could control the stress response and improve anxiety symptoms by restoring the gut microbiota (Liu et al., 2015). Our further work will test whether probiotics could improve the abnormal behaviors. At present, two major types of probiotics are commonly used: *Bifidobacterium* and *Lactobacillus* (Logan and Katzman, 2005; Rao et al., 2009; Silk et al., 2009). According to Wang et al., *Lactobacillus fermentum* strain NS9 administration not only normalized the composition of gut microbiota but reduced the anxiety-like behavior induced by ampicillin (Wang et al., 2015). Furthermore, the antidepressant effect of *Bifidobacterium infantis* has also been identified in the rat separation model of depression (Desbonnet et al., 2010).

Immune dysregulation was demonstrated by high levels of serum cytokines. This is supported by the evidence that inflammatory factors associate with a profile of behavioral changes (Capuron and Miller, 2011; Salim et al., 2012; Felger and Lotrich, 2013). Vagal sensory neurons express receptors for cytokines, so the inflammatory factors could directly activate the vagal afferents (Reardon et al., 2018). One study proposes that anxiety is related to inflammation; for example, mice infected with *Schistosoma mansoni* showed a reduction in behaviors such as exploration and grooming (Sulaiman et al., 1989). In addition, abnormal emotions, such as anxiety and neophobia, could happen following bacterial infection or as a response to bacterial products (Capuron and Miller, 2011). Anxiety levels increased when humans were exposed to lipopolysaccharide (LPS) (Grigoleit et al., 2011). Of the numerous cytokines, IL-6 is perceived as an atypical proinflammatory cytokine, having been demonstrated to show elevated levels in depressed animals and patients (Jiang et al., 2020; Lamers et al., 2020). In a study, IL-6 knockout mice became resistant to the development of depression-like symptoms (Monje et al., 2011). The underlying mechanisms involve in two pathways, the HPA axis and neurotransmitter metabolism, both of which are affected by increased IL-6 in depression (Ting et al., 2020). Furthermore, anxious patients also had higher serum levels of IL-6 than common people (Tang et al., 2018; Zou et al., 2020). In addition to impact on HPA axis activity, IL-6 could cross the blood–brain barrier, as they affect the uptake and release of mood-relevant neurotransmitters, including dopamine, 5-HT, noradrenaline, and gamma-aminobutyric acid (Zalcman et al., 1994; Clement et al., 1997; Anisman et al., 2008; Miller, 2009). IL-10, a prototypical anti-inflammatory cytokine, was closely related to depression (Li et al., 2020). Lower IL-10 has been observed in depression, while IL-10 was elevated after antidepressant treatment (Dai et al., 2020; Lee et al., 2020). In contrast, studies have reported higher IL-10 in depressive patients and decreased IL-10 after treatment for depression (Köhler et al., 2018; Himmerich et al., 2019; Wang et al., 2019; Brunoni et al., 2020). One explanation for increased IL-10 is that it is an anti-inflammatory response to correct an inflammatory activation caused by higher levels of proinflammatory cytokines (Bhattacharya and Drevets, 2017). In this way, higher IL-10 levels, as observed in our study, may be associated with the development of abnormal patterns. On the other hand, previous studies found a high dose of IL-10 may induce anxiety in the OFT (Harvey et al., 2006), two other behavioral tests for anxiety detection (Munshi et al., 2019). Taken together with these experiment evidences, the abnormal pattern of mice may be a direct result of increased inflammatory mediators.

Elevated corticosterone, one marker of HPA axis activation, was observed in the mice of the AB group (Borrow et al., 2019). Several studies indicate that the disturbance of gut bacteria affects the HPA axis. Specifically, adrenocorticotrophin and corticosterone levels for GF mice were higher than of mice bearing conventional microbiota (Crumeyrolle-Arias et al., 2014). Besides, the hyperactivity of the HPA response in GF mice could be partially reversed by gut microbiota transplantation (Huo et al., 2017). Probiotics, such as *Bifidobacterium* species, have been demonstrated to be efficient in restoring HPA axis function (Moya-Pérez et al., 2017).

The BDNF level showed a decreasing trend in the hippocampus of the AB group. According to the previous study, BDNF can maintain and promote development, differentiation and regeneration of neurons as well as affect learning and memory (Bercik et al., 2011). The hippocampus provides the brain with a spatiotemporal framework within which various sensory, emotional, and cognitive components are integrated (Yang and Wang, 2017). Literature has reported that hippocampus degeneration with diminished BDNF leads to a decline in cognition (Deltheil et al., 2008). Recently, gut microbiota is thought to directly affect BDNF expression. The GF mice showed a decreased BDNF in the cortex and hippocampus (Bercik et al., 2011). This coincides with the thesis that reduction of BDNF after gut dysbiosis possibly leads to impairment of cognitive function (Frohlich et al., 2016). Contrary to the findings, some studies observed an increased BDNF in the amygdala and hippocampus when gut microbiota imbalance induced a decline in spatial memory (Desbonnet et al., 2015). In addition, *Bifidobaterium adolescentis* shows a promising anxiolytic and antidepressant property as it up-regulated BDNF expression by restoring the balance of gut microbiota (Guo Y. et al., 2019). A slight increase of c-Fos was observed in the amygdala of the AB group. C-Fos serves as a component of transcription factor AP-1 and biomarker of neuronal activation, playing a major role in processing emotion and motivation (Baulmann et al., 2000; Roberts et al., 2019). Abnormal activation of c-Fos in the brain may be related to gut disorders; for example, as compared to uninfected mice, a significantly increased c-Fos was observed in mice infected with *Campylobacter jejuni* (Goehler et al., 2008). Meanwhile, a study indicated c-Fos activation following immune activation; this finding was in accord with our findings that cytokines increased with increasing c-Fos (Lyte et al., 2006).

## Conclusion

In general, we found that mice exposed to 11 weeks of ceftriaxone sodium treatment had a lower diversity and abundance of gut microbiota and showed more behavioral changes as compared to mice that were given normal saline. Dysregulation of the nerve-endocrine-immunological network may be a potential mechanism underlying abnormal behaviors induced by impaired gut microbiota. The study revealed the unknown side effects of antibiotics to a certain extent. Follow-up studies rebalancing the gut dysbacteriosis are required to further confirm the relationship between gut microbiota and brain function.

## Data Availability Statement

The datasets generated for this study can be found in the NCBI, SRA accession: PRJNA592623.

## Ethics Statement

The animal study was reviewed and approved by Animal Care Advisory Committee of Sichuan University.

## Author Contributions

ZZ and HY put forward the hypothesis, and BW, CT, LZ, and ML guided and supervised the experimental investigation and practices. LM, HW, and JL were responsible for data collection and analysis. YY provided pathologic diagnosis. ZZ was the main executer and involved in the entire research process, from proposal, planning and execution to implementation and composition. All authors contributed to the article and approved the submitted version.

## Conflict of Interest

The authors declare that the research was conducted in the absence of any commercial or financial relationships that could be construed as a potential conflict of interest.

## Supplementary Material

Supplementary Figure 1 Behavioral data analysis for eleven weeks. **(A)** Data from the open field test and **(B)** tail suspension test. AB: antibiotic group (*n* = 20), CT: control group (*n* = 20). Four mice of AB group were kicked out at the tenth week of gavage due to serious injuries influencing mobility.

## Abbreviations

MGB: microbiota-gut-brain
SPF: specific-pathogen-free
OFT: open-field test
TST: tail-suspension test
OTUs: operational taxonomic units
ELISA: enzyme-linked immunosorbent assay
BDNF: brain-derived neurotrophic factor
IL: interleukin
HPA: hypothalamic-pituitary-adrenal
CNS: central nervous system
BBB: blood–brain barrier
LPS: lipopolysaccharide
GF: germ-free.
