# Supplementary figures and images for: Long-Term Exposure to Ceftriaxone Sodium Induces Alteration of Gut Microbiota Accompanied by Abnormal Behaviors in Mice
Source: Front Cell Infect Microbiol. 2020 Jun 24;10:258. doi: 10.3389/fcimb.2020.00258 (PMC7344183; doi:10.3389/fcimb.2020.00258)

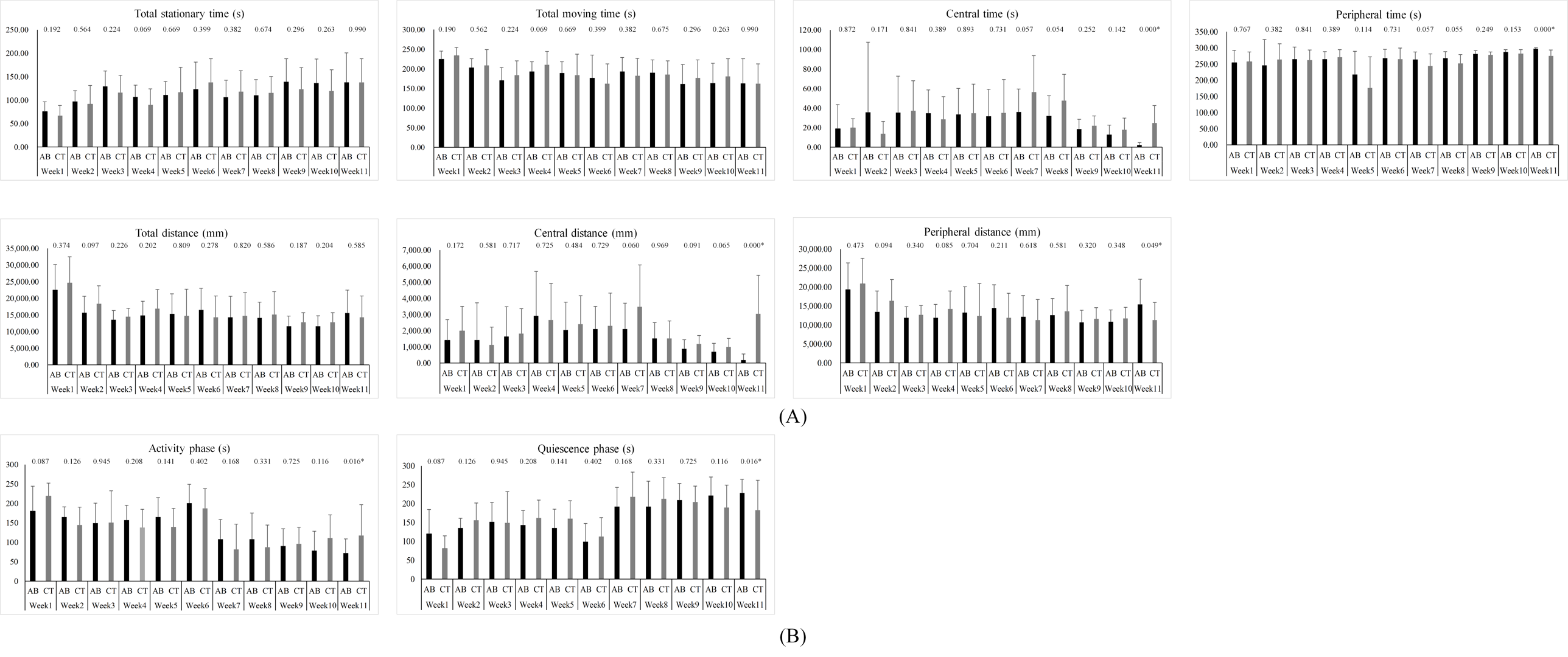

Supplement: Supplementary Figure 1 — Behavioral data analysis for eleven weeks. (A) Data from the open field test and (B) tail suspension test. AB: antibiotic group (n = 20), CT: control group (n = 20). Four mice of AB group were kicked out at the tenth week of gavage due to serious injuries influencing mobility. [file Image_1.tif]
