# Supplementary material for: Long-Term Exposure to Ceftriaxone Sodium Induces Alteration of Gut Microbiota Accompanied by Abnormal Behaviors in Mice
Source: Front Cell Infect Microbiol. 2020 Jun 24;10:258. doi: 10.3389/fcimb.2020.00258 (PMC7344183; doi:10.3389/fcimb.2020.00258)
Supplement: Supplementary file 2 [file Data_Sheet_1.docx]

**Supplementary materials**

**Determining of the ceftriaxone sodium dosage in preliminary experiment**

The ceftriaxone sodium dosage was based on preliminary experiments to determine maximum tolerable dosage. According to previous study, 12 mice were treated with 0.2 ml different doses of ceftriaxone sodium (100, 200 and 400 mg/mL) intragastrically (Guo et al. 2017). After one week of treatment, a higher concentration of the antibiotic led to excessively insubordinate behavior, and even 2 mice in 400 mg/mL group died. But, mice in other two groups had no abnormalities. Considering the dose of 100mg/ml may require longer periods of observation, the dose of gavage administration was adjusted to slightly more than 200mg/ml.

Reference:

Guo Yanjie,Yang Xuefei,Qi Yane et al. Long-term use of ceftriaxone sodium induced changes in gut microbiota and immune system.[J] .Sci Rep, 2017, 7: 43035.
